# Mechanisms of Diesel-Induced Endothelial Nitric Oxide Synthase Dysfunction in Coronary Arterioles

**DOI:** 10.1289/ehp.1002286

**Published:** 2010-09-22

**Authors:** Tom W. Cherng, Michael L. Paffett, Olan Jackson-Weaver, Matthew J. Campen, Benjimen R. Walker, Nancy L. Kanagy

**Affiliations:** 1 Department of Cell Biology and Physiology and; 2 Department of Pharmaceutical Sciences, University of New Mexico, Health Sciences Center, Albuquerque, New Mexico, USA

**Keywords:** arteries, cardiovascular, engine emissions, exhaust, nitric oxide synthase, *N*^ω^-nitro-l-arginine, particulate matter, rat

## Abstract

**Background and objective:**

Increased air pollutants correlate with increased incidence of cardiovascular disease potentially due to vascular dysfunction. We have reported that acute diesel engine exhaust (DE) exposure enhances vasoconstriction and diminishes acetylcholine (ACh)-induced dilation in coronary arteries in a nitric oxide synthase (NOS)-dependent manner. We hypothesize that acute DE inhalation leads to endothelial dysfunction by uncoupling NOS.

**Methods:**

Rats inhaled fresh DE (300 μg particulate matter/m^3^) or filtered air for 5 hr. After off-gassing, intraseptal coronary arteries were isolated and dilation to ACh recorded using videomicroscopy.

**Results:**

Arteries from DE-exposed animals dilated less to ACh than arteries from air-exposed animals. NOS inhibition did not affect ACh dilation in control arteries but increased dilation in the DE group, suggesting NOS does not normally contribute to ACh-induced dilation in coronary arteries but does contribute to endothelial dysfunction after DE inhalation. Cyclooxygenase (COX) inhibition did not affect ACh dilation in the DE group, but combined inhibition of NOS and COX diminished dilation in both groups and eliminated intergroup differences, suggesting that the two pathways interact. Superoxide scavenging increased ACh dilation in DE arteries, eliminating differences between groups. Tetrahydrobiopterin (BH_4_) supplementation with sepiapterin restored ACh-mediated dilation in the DE group in a NOS-dependent manner. Superoxide generation (dihydroethidium staining) was greater in DE arteries, and superoxide scavenging, BH_4_ supplementation, or NOS inhibition reduced the signal in DE but not air arteries.

**Conclusion:**

Acute DE exposure appears to uncouple NOS, increasing reactive oxygen species generation and causing endothelial dysfunction, potentially because of depletion of BH_4_ limiting its bioavailability.

Exposure to vehicular pollutants is associated with exacerbation of both cardiovascular and respiratory diseases (Ibald-Mulli et al. 2002; Peters et al. 2004; Pope 2000; Timonen et al. 2006; Wichmann et al. 2000). Diesel engine exhaust (DE) is an important contributor to urban air pollution (Brunekreef and Holgate 2002; Laden et al. 2000; Pope et al. 1999). Although the exact components of DE responsible for its effects have yet to be defined, both short-term and chronic DE exposure are associated with arrhythmias and adverse cardiac events (Dockery et al. 2005; Peters et al. 2000; Rich et al. 2005) and cause endothelial dysfunction that diminishes vasodilator response in systemic arteries (Hansen et al. 2007). Multiple pathways regulate endothelium-dependent vasodilatation, including the release of nitric oxide (NO) and prostacyclin from the nitric oxide synthase (NOS) and cyclooxygenase (COX) pathways, respectively. Additionally, activation of small (SK) and intermediate (IK) Ca^2+^-dependent potassium channels can result in the hyperpolarization and ultimately the relaxation of vascular smooth muscle. However, short-term inhalation of dilute DE has been shown to inhibit forearm vasodilation to both acetylcholine (ACh) and the NO donor, sodium nitroprusside, in healthy volunteers (Mills et al. 2005; Tornqvist et al. 2007), suggesting that DE exposure may specifically affect signaling downstream of NO.

Endothelial NOS (eNOS) is a homodimeric protein that generates NO from the conversion of l-arginine to l-citrulline. NO inhibits platelet aggregation, leukocyte adherence, and vascular smooth muscle proliferation to regulate vascular homeostasis (Alheid et al. 1987; Kubes et al. 1991; Nunokawa and Tanaka 1992). Synthesized NO diffuses into adjacent vascular smooth muscle, where it activates soluble guanylate cyclase to reduce intracellular Ca^2+^ concentration and decrease vascular tone, leading to vasodilation. The eNOS cofactor 5,6,7,8-tetrahydrobiopterin (BH_4_) is required for the production of NO, possibly by stabilizing the physical and/or electrochemical coupling of the NOS dimer, and is generated from guanosine-5′-triphosphate (GTP) through a *de novo* pathway or recycled from the oxidized form of BH_4_, 7,8-dihydrobiopterin (BH_2_) by dihydrofolate reductase (DHFR) (Sugiyama et al. 2009).

DE exposure increases oxidative stress within the vasculature (Miller et al. 2009), which can potentially increase the oxidation of BH_4_ to BH_2_, thereby limiting the bioavailability of this essential cofactor. Depletion of BH_4_ or increases in BH_2_ electrochemically uncouple eNOS, resulting in the generation of superoxide radical (O_2_^−^) rather than NO (Crabtree et al. 2008; Sugiyama et al. 2009). In a cascade fashion, peroxynitrite (ONOO^−^) production from the reaction of O_2_^−^ and NO not only depletes bioavailable NO, but can also oxidize BH_4_ to catalyze further eNOS uncoupling. We have previously reported that vasoconstriction is augmented in systemic arteries from DE-exposed rodents and that the augmented constriction is endothelium dependent and can be reversed with NOS inhibition (Campen et al. 2005; Cherng et al. 2009; Knuckles et al. 2008). The aim of this study was to evaluate agonist-mediated vasodilation in coronary arteries from healthy rats exposed to DE. We hypothesized that DE-induced reactive oxygen species (ROS) uncouple NOS to diminish NO-dependent vasodilatation in coronary arteries.

## Methods

### Animals

All animal protocols were reviewed and approved by the Institutional Animal Care and Use Committee of the Lovelace Respiratory Research Institute (LRRI) and the University of New Mexico and conform to National Institutes of Health guidelines for animal use to ensure animals were treated humanely and with regard for the alleviation of suffering (Institute of Laboratory Animal Resources 1996). Male Sprague-Dawley rats (250–300 g; Charles River Laboratories, Portage, MI) were housed in quarantine for 14 days after receipt, then acclimated to exposure chambers for 7–14 days. Within 2-m^3^ stainless steel and plexiglas chambers (Hazleton Systems, Maywood, NJ), rats were housed in standard shoebox cages and maintained on a 12:12 hr light:dark cycle, with food and water available *ad libitum* before exposures. Food was withdrawn during DE exposure.

### DE exposure

Rats were exposed to 300 μg particulate matter (PM)/m^3^ DE in a sealed chamber for 5 hr representing the daily PM exposure limit set by theU.S. Environmental Protection Agency (2010). Although daily exposure in most of the U.S. population is far lower in terms of PM, occupations requiring the use of diesel engines have exposure conditions similar to this study (Pronk et al. 2009). The DE system has been previously characterized and produces levels of CO and nitrogen oxide (NO_x_) at approximately 3 ppm and 4 ppm, respectively (McDonald et al. 2004). Control (Air) rats were housed identically but exposed to filtered air. DE was generated from a single-cylinder, 5,500-watt, Yanmar diesel generator (Yanmar, Adairsville, GA) using nationally certified diesel fuel at the LRRI facility. Electrical current was drained from the engine to provide a constant 90% load during operation to ensure consistent emissions. The particle concentration was monitored by sampling on 47-mm Pallflex (Pall-Gelman, Port Washington, NY) filters. Filters were collected twice per day (every 3 hr) for each DE exposure chamber and once per day from the control chamber. Prefilter and postfilter weights were measured with a microbalance, and desired concentrations of the emissions were attained by diluting the direct exhaust with filtered air. Exposure chamber temperature and humidity were monitored throughout exposures, and temperatures were maintained at 20–25°C (McDonald et al. 2004).

### Isolated artery preparation

At the end of the 5-hr exposure, chambers were off-gassed for 30 min, and the rats were removed and euthanized with sodium pentobarbital (200 mg/kg, intraperitoneal). Hearts were immediately removed and intraseptal coronary arteries (resting inner diameter, Air: 176 ± 7 μm, DE: 180 ± 7 μm) were isolated and placed in chilled physiological saline solution (PSS, in micromoles per liter; 129.8 NaCl, 5.4 KCl, 0.83 MgSO_4_, 19 NaHCO_3_, 1.8 CaCl_2_, and 5.5 glucose) aerated with 21% O_2_, 6% CO_2_, and 73% N_2_. Each artery was used for only one experimental protocol. Both ends of the arteries were cannulated onto glass micropipettes in a tissue chamber (Living Systems, CH-1; LSI, Burlington, VT) and secured with silk sutures within 30 min of isolation from the heart. Vessels were stretched to approximate *in situ* length and pressurized to 60 mmHg with PSS in the lumen absent of flow and superfused at a rate of 5 mL/min with 37°C oxygenated PSS. At the end of the experiment, Ca^2+^-free PSS (3.7 mmol/L EGTA) was superfused for 60 min to fully relax the vessel (Ca^2+^-free inner diameter, Air: 219 ± 4 μm, DE: 224 ± 4 μm).

### Vasodilator studies

Vessel chambers were placed on the stage of an inverted Nikon Eclipse TS 100 (Nikon Instruments, Irvine, CA) microscope fitted with a video camera connected to a data acquisition computer. Inner diameter changes were recorded using edge detection software (IonOptix, Milton, MA) as described previously (Duling et al. 2006). Arteries were treated for at least 30 min with various drug treatments or vehicle (Veh) in the superfusate and in the lumen before dilation with increasing concentrations of ACh (0.001–100 μmol/L) in U46619 (a thromboxane mimetic) preconstricted arteries (constricted to ~ 50% of fully relaxed diameter). The contribution of key endothelial dilator pathways was determined using NOS inhibitor [*N*^ω^-nitro-l-arginine (l-NNA), 100 μmol/L], COX inhibitor (indomethacin or aspirin, 10 μmol/L), and small (apamin, 100 nmol/L) and intermediate (Tram-34, 1 μmol/L) Ca^2+^-activated K^+^ channels. The role of NOS uncoupling and ROS-mediated endothelial dysfunction after exposure was assessed with BH_4_ donor (sepiapterin, 1 μmol/L) and the cell permeate superoxide dismutase (PEG-SOD, 150 U/mL), respectively.

### Quantitative real-time polymerase chain reaction analysis

RNA from intraseptal coronary artery homogenate was isolated using Qiagen RNeasy Fibrous Tissue Mini Kit (Qiagen USA, Valencia, CA) according to manufacturer’s protocol. Reverse transcriptase synthesis of cDNA and real-time quantitative polymerase chain reaction (qPCR) were performed as described elsewhere (de Frutos et al. 2008). In brief, multiplex qPCR amplification of TaqMan probe sets (Applied Biosystems, Carlsbad, CA) for DHFR and GTP cyclohydrolase (GTPCH) with respect to β-actin was performed using a 7500 Fast Real-Time PCR System (Applied Biosystems). Relative expression (^ΔΔ^C_T_) was calculated by subtracting the C_T_ of the endogenous β-actin control gene from the C_T_ value of the gene of interest where the normalized gene expression method (2^−ΔΔ^*^C^T^^*) was used for relative quantification of gene expression (Livak and Schmittgen 2001).

### ROS measurement

Oxidation of the cell-permeable fluorescent probe dihydroethidium (DHE) by O_2_^−^ generates ethidium^+^, which then intercalates into DNA of cells (Zhao et al. 2003, 2005). Septum from both Air- and DE-exposed animals were embedded in O.C.T. Compound (TissueTek; Sakuta Fineteck USA, Torrence, CA) without fixation and flash-frozen with liquid nitrogen. Septal sections (10 μm) were allowed to dry on glass slides for 30 min at room temperatures preceded by treatment with phosphate-buffered saline (PBS), l-NNA (100 μmol/L), sepiapterin (1 μmol/L), or the superoxide scavenger tiron (10 μmol/L) for 30 min at 37°C, followed by incubation with DHE (10 μmol/L) with inhibitors for 45 min at 37°C. Cover slips were mounted on each slide with Vectashield (Vector Laboratories, Burlingame, CA). Images of coronary arteries were captured on a Nikon Optiphot fluorescence microscope (Nikon Instruments) using a Chroma TRITC filter set (Chroma Technology Corp, Bellows Falls, VT) (excitation: 510–560 nm; emission: 570–650 nm). Data are expressed as average intensity (integrated intensity per area of region of interest) from one artery per rat for six rats in each group and were gathered using Metamorph software (version 7.0; Molecular Devices, Sunnyvale, CA).

### Statistical analysis and calculations

Dilator responses were analyzed using two-way repeated-measures analysis of variance (ANOVA) with Student–Newman–Keuls post hoc analysis (Sigma Stat, version 3.5; Systat Software, Inc., San Jose, CA) for differences between groups, concentrations, and interactions. Probability levels < 0.05 were considered significant. Data were tested for normality as part of the two-way ANOVA analysis. All dilation data are expressed as a percent reversal of active tone (50% U46619 preconstricted) and is calculated as the percent change in inner diameter as shown below.


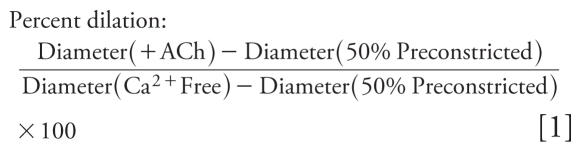


## Results

### DE inhibits ACh-mediated vasodilatation

**Figure 1:**
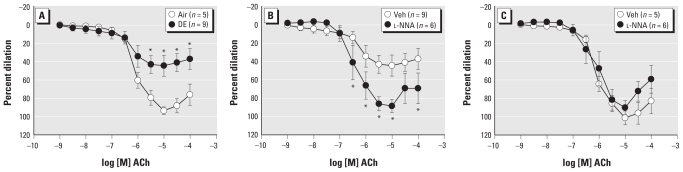
ACh vasodilation was diminished after exposure to DE compared with Air control in vehicle (Veh)-treated coronary arteries (*A*). NOS inhibition with l-NNA (100 μmol/L) restored the blunted ACh-mediated dilation (*B*) without affecting dilation in the Air group (*C*). *p < 0.05 compared with Air or Veh.

First, we determined whether diesel exposure affected endothelium-dependent responses to ACh. Coronary arteries from DE-exposed rats (*n* = 9) had diminished vasodilation to ACh compared with arteries from air-exposed rats (*n* = 5, *p =* 0.006, Figure 1A). Maximal agonist-induced dilation in Air (94 ± 4%) was greater than DE arteries (44 ± 12%, *p =* 0.007). In Ca^2+^-free PSS, the inner diameter of fully relaxed coronary arteries was not different between Air and DE groups (200 ± 6 μm vs. 210 ± 13 μm, respectively). These findings note a substantial reduction in dilatory response after a single acute exposure to DE.

### NOS, but not COX, inhibition restores dilation after DE

**Figure 2:**
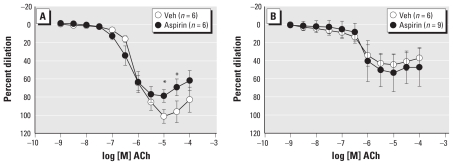
Pretreating coronary arteries with aspirin (10 μmol/L) to inhibit COX diminished ACh-mediated dilation in the Air group (*A*) but had no effect in the DE-exposed group (*B*) compared with vehicle (Veh) treatment. *p < 0.05 compared with Veh.

To elucidate which endothelium-dependent pathways were altered by DE exposure, we investigated the roles of NOS, COX, and SK and IK channels. NOS inhibition (*n* = 6) augmented ACh-mediated dilation in the DE group compared with vehicle treatment (*p =* 0.011, Figure 1B), restoring dilation to that in untreated Air arteries (*p =* 0.945), suggesting an NOS-dependent inhibition of dilation. Unexpectedly, l-NNA (100 μmol/L) did not alter dilation in the Air group compared with untreated controls (*p =* 0.456, Figure 1C). In contrast, COX inhibition with aspirin (10 μmol/L) modestly inhibited dilation in the Air arteries (*n* = 6, *p =* 0.040, Figure 2A), but did not affect ACh-mediated responses in the DE group (*n* = 9, Figure 2B).

**Figure 3:**
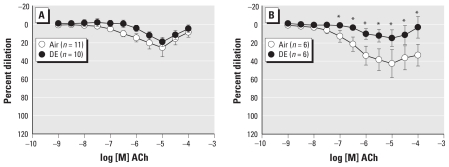
Inhibition of both NOS (l-NNA, 100 μmol/L) and COX (aspirin, 10 μmol/L) diminished dilation to ACh in both groups and eliminated between-groups differences (*A*). Although blockade of small and intermediate Ca^2+^-dependent K^+^ channels blunted dilation in arteries from both groups, dilation was still less in the DE-exposed compared with the Air group (*B*). *p < 0.05 compared with Air.

The combined inhibition of NOS and COX blunted ACh-mediated dilation in both DE and Air group (*n* = 10 and *n* = 11, respectively, Figure 3A) compared with vehicle treatment within group. Blockade of SK and IK with Tram-34 (100 nmol/L) and apamin (1 μmol/L), respectively, diminished ACh-mediated dilation significantly and similarly in both groups (*n* = 6 per group, Figure 3B) compared with vehicle treatment, but the residual dilation was less in DE than in Air arteries. Finally, combined inhibition of NOS, COX, SK, and IK completely abolished dilation in both groups (data not shown). Although inhalation of DE alters other endothelium-dependent dilator pathways, the impairment of NOS function, primarily, results in diminished ACh-mediated dilation.

### Diminished dilation after DE is restored with BH_4._

**Figure 4:**
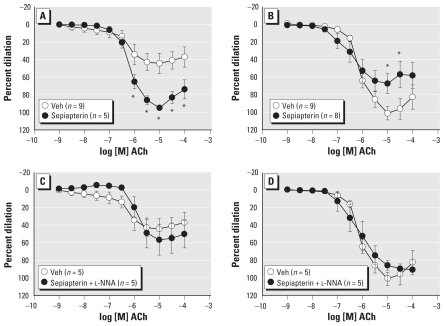
Dilation to ACh was augmented in arteries from the DE group after BH_4_ supplementation (sepiapterin, 1 μmol/L) compared with vehicle (Veh) treatment (*A*). In the presence of NOS inhibition (l-NNA, 100 μmol/L), the effect of sepiapterin was blocked (*B*). Dilator response to ACh was blunted in arteries from the Air group after BH_4_ supplementation compared with Veh treatment (*C*). In the presence of NOS inhibition, the effect of sepiapterin was blocked (*D*). *p < 0.05 compared with Veh.

To determine whether supplementation with the cofactor BH_4_ could rescue NOS function, arteries were treated with sepiapterin (1 μmol/L), which increases BH_4_ levels through the salvage pathway, via DHFR, of BH_4_ biosynthesis. Sepiapterin treatment of isolated arteries augmented and restored ACh-mediated dilation in DE arteries (*n* = 5, Figure 4A) while blunting dilation in the Air group (*n* = 8, Figure 4C). The effects of sepiapterin on dilation were blocked by concurrent NOS inhibition (*n* = 5 per group, Figures 4B and 4D) such that there was no difference from vehicle treatment within each group. After exposure, BH_4_ levels appear to be insufficient to maintain NOS function.

The reduction in BH_4_ levels may result from a decrease in the BH_4_ biosynthesis pathway after DE exposure. A decrease in GTPCH would blunt *de novo* synthesis of BH_4_ whereas inhibition of DHFR reduces the recovery of BH_2_ back to BH_4_. Quantitative real-time PCR revealed that DE exposure (*n* = 7) does not impact intraseptal coronary artery mRNA expression of GTPCH (*p =* 0.797) or DHFR (*p =* 0.558; data not shown) compared with Air (*n* = 8) exposure.

### Superoxide scavenging prevents effects of DE exposure

**Figure 5:**
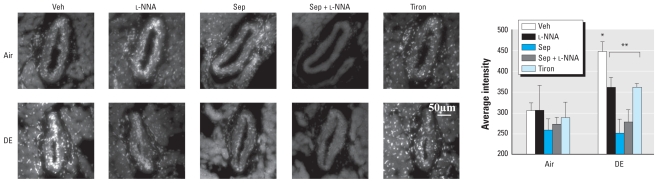
DHE fluorescence was greater in PBS-treated (Veh) coronary arteries from DE-exposed compared with Air-exposed rats. Tiron (10 μmol/L), l-NNA (100 μmol/L), sepiapterin (1 μmol/L), or sepiapterin with l-NNA treatment *ex vivo* prevented the DE-induced increase in fluorescence without effect in Air arteries. *p < 0.05 compared with Air Veh. **p < 0.05 compared with DE Veh (*n* = 6/group).

**Figure 6:**
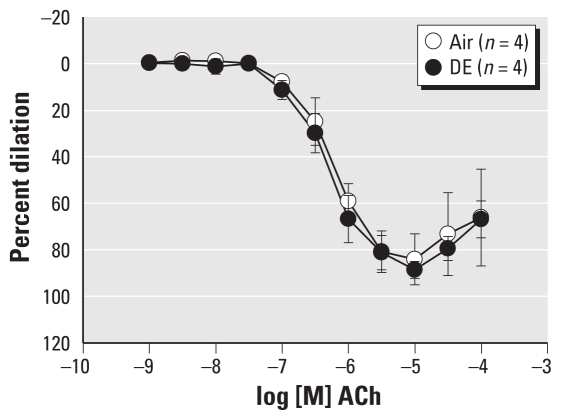
Scavenging of superoxide with PEG-SOD (150 U/mL) restored the dilator response to ACh in the DE group but did not affect responses in the Air group.

DE exposure can increase oxidative stress, which can reduce both NO and BH_4_ levels (Katusic 2001; Miller et al. 2009). Coronary artery superoxide levels were evaluated with the cell-permeable fluorescent probe DHE. Septal coronary arteries from rats exposed to DE induced greater DHE fluorescence than arteries from Air-exposed rats. Scavenging O_2_^−^ with tiron or inhibition of NOS had no effect on DHE fluorescence in Air, but diminished and normalized fluorescence in DE compared with Air (*n* = 6 per group) (Figure 5), indicating increased O_2_^−^ generation after DE exposure that is NOS dependent. BH_4_ supplementation in the presence or absence of NOS inhibition similarly diminished DHE fluorescence only in DE coronary arteries, reducing it to the levels seen in Air arteries and supporting the conclusion that BH_4_ bioavailability may be depleted after DE exposure. ROS were scavenged with SOD to determine if oxidative stress mediates the vascular changes of DE exposure. Treatment of Air arteries with PEG-SOD (150 U/mL) had no effect on ACh-mediated vasodilation compared with vehicle treatment. Similar to NOS inhibition or BH_4_ supplementation, superoxide scavenging with PEG-SOD restored dilation in the DE group so that dilation was not different from arteries in the Air vehicle treatment group (*n* = 4 per group) (Figure 6). Exposure to DE appears to augment superoxide generation that inhibits agonist-induced dilation.

## Discussion

In our study, acute exposure to DE impaired ACh-mediated dilation in coronary arteries from healthy rats, an effect that was reversed by NOS inhibition. Supplementation with the BH_4_ precursor sepiapterin or scavenging ROS *in vitro* also completely restored ACh-mediated dilation after DE exposure. Although COX inhibition did not alter dilation in either group, combined blockade of NOS and COX significantly blunted dilation in arteries from both Air and DE-exposed animals. Additional inhibition of SK and IK channels completely abolished dilation, with no difference between DE and filtered air groups. Combined, these observations suggest that DE exposure diminishes ACh-mediated dilation by selectively disrupting NOS-mediated responses.

The most likely mechanism for loss of ACh-induced dilation in the DE arteries is oxidative stress-induced uncoupling of NOS. As sepiapterin supplementation antagonized the effects of DE, the current results suggest that NOS may be uncoupled because of a loss of BH_4_. Uncoupled NOS generates O_2_^−^ rather than NO, which can further scavenge BH_4_ (Katusic 2001; Munzel et al. 2005; Nurkiewicz et al. 2010). BH_4_ is necessary for physical and electrochemical coupling of NOS, and the depletion of this cofactor in an oxidative environment further exacerbates NOS and endothelial dysfunction. In this study, mRNA levels of GTPCH and DHFR were not altered by DE exposure, suggesting the *de novo* and salvaging pathways of BH_4_ biosynthesis were not impaired. However, supplementation with sepiapterin, a precursor to BH_4_, fully restored ACh-mediated dilation in coronary arteries from the DE-exposed group, suggesting that loss of this cofactor led to the impaired dilation.

Inhibition of NOS in DE-exposed rats prevented the effects of sepiapterin, suggesting the effect of BH_4_ was to recouple NOS. In contrast, BH_4_ supplementation in coronary arteries from Air-exposed animals had the opposite effect, diminishing endothelium-mediated dilation. These effects also appear to be NOS dependent, as treatment with l-NNA prevented sepiapterin blunting of dilation in the Air arteries. Sepiapterin increases levels of BH_2_, the oxidized form of BH_4_, which is converted to BH_4_ by dihydrofolate reductase through the salvage pathway (Thony et al. 2000). If the salvage pathway is saturated by excess BH_2_ levels, sepiapterin release of BH_2_ can inhibit NOS function by competing with BH_4_ for NOS binding to mimic BH_4_ depletion and increase ROS production by uncoupling NOS (Crabtree et al. 2008; Sugiyama et al. 2009). In this manner, sepiapterin may generate an oxidative environment in the Air arteries that is partially NOS driven, similar to the effects of DE exposure. Inhibition of NOS in arteries from Air-exposed animals treated with sepiapterin blunts ROS generation and restores endothelial function.

Basal levels of superoxide detected by DHE staining in coronary arteries were elevated after DE exposure compared with the Air group. This increase in superoxide generation was further indicated by the increased tiron-sensitive signal that was blocked with NOS inhibition or BH_4_ supplementation. These data suggest DE exposure uncouples NOS by decreasing BH_4_ levels to augment ROS generation. NOS inhibition in the presence of sepiapterin was not different from sepiapterin alone, which suggests that BH_4_ supplementation restored NOS function to a level that could be inhibited with l-NNA. It was recently described that the specificity of DHE fluorescence may not represent the intracellular levels of superoxide accurately using fluorescent microscopy (Zielonka and Kalyanaraman 2010). Nonetheless, our study attempted to address the flaws of this methodology by comparing the component sensitive to tiron, a superoxide scavenger, rather than the raw values. Further supporting that DE effects were mediated by elevated levels of ROS, scavenging ROS with PEG-SOD in coronary arteries from DE-exposed animals restored agonist-mediated dilation but did not alter dilation in the Air group. SOD catalyses the conversion of O_2_^−^ to hydrogen peroxide, which is further broken down to H_2_O and O_2_ by catalase. The restoration of ACh-induced dilation with PEG-SOD may be partially mediated by increased H_2_O_2_, which has been shown to be a vasodilator (Miura et al. 2003). However, taken with the observations that NOS inhibition and BH_4_ supplementation also restore endothelial function, the present data are more consistent with the conclusion that after DE inhalation, uncoupled NOS generates O_2_^−^. The increase in O_2_^−^ depletes bioavailability of both NO and BH_4_ to potentiate further uncoupling of NOS. In contrast to our findings, Courtois et al. (2008) found NO-dependent relaxation was impaired after intratracheal PM instillation secondary to inflammation that decreased smooth muscle sensitivity to NO independent of ROS generation. As previously reported, acute inhalation of DE at moderate levels (i.e., 300 μg/m^3^) does not induce measureable changes in pulmonary inflammatory markers nor does it alter vascular smooth muscle sensitivity to basal NO (Cherng et al. 2009). A major difference between our study design and that of Courtois et al. (2008) as well as Nurkiewicz et al. (2006) is that we investigated effects immediately after exposures rather than 6–72 hr later. Thus, inflammatory contributions to the systemic vasculature are minimal in our model, as evidenced by lack of cytokine induction, whereas at later time points, evidence of rolling and adhering leukocytes and dexamethasone-sensitive vascular impairments can be seen. The differences in exposure method, instillation of urban PM versus inhalation of dilute whole DE, and of vascular bed of interest, pulmonary versus systemic, between Courtois et al. and the present study may have led to the different mechanisms of NO impairment observed in these two studies. In fact, these differences provide insight into the multiple mechanisms leading to endothelial dysfunction after inhaled versus instilled exposure to air pollution.

Contrary to our hypothesis that sepiapterin treatment in AIR arteries augments BH_2_ levels to uncouple NOS, DHE fluorescence was not increased by addition of the BH_2_ donor in Air arteries. It has been demonstrated that BH_4_ is a weak antioxidant and that this may partially mediate its ability to restore endothelial function (Vasquez-Vivar et al. 2002). Therefore, sepiapterin treatment may have elevated BH_2_ levels in the AIR arteries to increase NOS production of O_2_^−^, which was then scavenged by BH_4_, so that DHE fluorescence appeared unchanged but ACh dilation was still impaired. Additional studies directly measuring NO production before and after BH_4_ treatment will be necessary to determine whether sepiapterin indeed impairs NOS function. Alternatively, sepiapterin-derived BH_4_ could facilitate NO synthesis to inhibit COX-dependent production of vasodilators to impair ACh-mediated dilation (Boulos et al. 2000). In this case, NOS inhibition would have decreased NO synthesis to restore COX-mediated dilation, similar to NOS inhibition alone in the Air arteries.

Interactions between the NOS and COX pathways have been demonstrated previously in both cultured cells and isolated arteries (Bratz and Kanagy 2004) and are apparent in the current study. In Air arteries, COX inhibitors only modestly diminished ACh-mediated dilation, whereas NOS inhibition had no effect; however, the combined inhibition greatly blunted agonist-induced dilation. These results suggest the two pathways exhibit functional redundancy such that pharmacological blockade of one pathway can be offset by activation through the alternate pathway. Thus, inhibition of both NOS and COX blunts dilation more than the sum of inhibiting either pathway alone. The synergistic effect of combined inhibition of NOS and COX was seen in both Air and DE arteries, suggesting that NOS/COX interaction is not lost after DE exposure and plays an essential role in the coronary artery.

Blockade of the SK and IK channels inhibited dilation in both groups, but dilation was still blunted in the DE compared with Air arteries in the presence of the inhibitors. Therefore, activation of SK and IK channels contribute to ACh-induced dilation but do not appear to be altered by DE exposure. Interpretation of these results is complicated, because these potassium channels regulate endothelial Ca^2+^ entry, which affects both NOS and COX activity. Inhibition of these channels induces membrane depolarization that reduces the electrochemical driving force for Ca^2+^ entry upon agonist stimulation (Faraci and Heistad 1998), potentially reducing both NOS and COX activation. In sum, the data show that ACh-induced dilation in coronary arteries is mediated by NOS, COX, SK, and IK, as combined inhibition of these pathways was necessary to completely abolish dilator responses.

Both human and animal studies suggest the NOS pathway is impaired by DE exposure, but mechanistic details have not been fully elucidated. Recently, Nurkiewicz and colleagues (2009) found that NO availability is reduced in vessels isolated from rats exposed acutely to ultrafine PM. However, it was not clear whether NOS was uncoupled or whether generated NO was scavenged; both mechanisms appear likely. Previously, we demonstrated that inhaled DE enhances endothelin-1 (ET-1) vasoconstriction in rat coronary arteries by stimulating uncoupled NOS-dependent constriction via endothelial endothelin B receptor (Cherng et al. 2009). Langrish et al. (2009) demonstrated similar findings in healthy human volunteers exposed to DE, where circulating ET-1 was not different but ET-1 constrictor sensitivity was enhanced, apparently via endothelin B receptor pathways. In the present study, DE exposure also diminishes ACh-mediated dilation, which was restored by NOS inhibition or BH_4_ supplementation, further supporting the conclusion that DE exposure uncouples eNOS. ROS scavenging also reversed the effects of DE inhalation, supporting a role for increased oxidative stress, which can decrease NOS mediated dilation through multiple pathways. Elevated ROS levels presumably oxidize BH_4_, thereby decreasing availability of this necessary cofactor for NO generation and resulting in NOS uncoupling. BH_4_ supplementation provides enough excess cofactor that NOS stays in a coupled state, preventing the effects of DE inhalation. Furthermore, NO can be scavenged by ROS to form ONOO^−^, which not only depletes NO bioavailability but can nitrosylate proteins to alter function (Alderton et al. 2001). Further studies are needed to determine if the initial source of ROS is DE or uncoupled NOS. It may be that DE provides the initial oxidative burst that leads to the uncoupling of NOS, resulting in a feed-forward cycle leading to endothelial dysfunction. Alternatively, DE may provide a substrate for NOS uncoupling that is not related to ROS.

It is clear that pharmacologically induced vasodilatation is blunted in healthy volunteers (Mills et al. 2005; Tornqvist et al. 2007) and in animal models (Cherng et al. 2009; Knuckles et al. 2008) after acute inhalation of DE. It is unclear whether the alterations in vascular function in the current study persist at later time points; future studies will address this issue. We speculate similar changes will be present but waning over the following 24–72 hr as the vascular alterations in humans, which have rapid onset (Mills et al. 2005) and parallel our observations in the coronary arteries, persist at least 24 hr after cessation of exposure (Tornqvist et al. 2007). Potentially, activation of uncoupled NOS after DE inhalation may result in prolonged endothelial dysfunction that is only slowly reversed after exposure. It is likely that vascular endothelium must recover by the *de novo* production of NOS protein along with cofactors such as BH_4_ which, depending on the potency of exposure, abundance of precursors and nutrients, and genetic factors, could take several days to recuperate. Furthermore, DE inhalation can increase circulating levels of the endogenous vasoconstrictor ET-1 in both animal (Vincent et al. 2001) and human (Peretz et al. 2008) studies and enhance vascular responsiveness to ET-1 (Cherng et al. 2009). Such changes would be expected to further diminish dilation and enhance constriction in coronary vessels, thereby increasing risk and/or severity of coronary occlusive sequelae. Thus, susceptible individuals with underlying cardiovascular conditions may have augmented coronary vasoconstriction after DE exposure, contributing to the increased cardiovascular events seen epidemiologically after elevated pollution levels.
